# A multinational cross-sectional study on the awareness and concerns of healthcare providers toward monkeypox and the promotion of the monkeypox vaccination

**DOI:** 10.3389/fpubh.2023.1153136

**Published:** 2023-05-09

**Authors:** Sarya Swed, Hidar Alibrahim, Haidara Bohsas, Nagham Jawish, Mohammed Amir Rais, Mohamad Nour Nasif, Wael Hafez, Bisher Sawaf, Ahmed Abdelrahman, Sherihan Fathey, Ismail Atef Ismail Ahmed Ibrahim, Sondos Hussein Ahmad Almashaqbeh, Razan Mohammad Yousef Aljawarneh, Amine Rakab, Emad Hassan Hassan EL-Shafei, Rene Hurlemann, Mohamed E. G. Elsayed, Data Collection Group, Albaraa Daradkeh

**Affiliations:** ^1^Faculty of Medicine, Aleppo University, Aleppo, Syria; ^2^Damascus University Faculty of Medicine, Damascus, Syria; ^3^Faculty of Medicine of Algiers, University of Algiers, Algiers, Algeria; ^4^New Medical Centre (NMC) Royal Hospital, Khalifa City, United Arab Emirates; ^5^Medical Research Division, Department of Internal Medicine, The National Research Centre, Cairo, Egypt; ^6^Department of Internal Medicine, Hamad Medical Corporation, Doha, Qatar; ^7^Internal Medicine Department, Faculty of Medicine Zagazig University, Zagazig, Egypt; ^8^Department of Health, Giza, Egypt; ^9^Physical Therapy and Rehabilitation, Fenerbahçe Üniversitesi, Istanbul, Türkiye; ^10^Faculty of Medicine, University of Jordan, Amman, Jordan; ^11^Faculty of Medicine, Jordan University of Science and Technology, Irbid, Jordan; ^12^Department of Clinical Medicine, Weill Cornell Medical College, Ar-Rayyan, Qatar; ^13^Critical Care Medicine, Mediclinic Alnoor Hospital, Abu Dhabi, United Arab Emirates; ^14^Department of Psychiatry, School of Medicine and Health Sciences, Carl von Ossietzky University Oldenburg, Oldenburg, Germany; ^15^Department of Psychiatry, University Hospital Bonn, Bonn, Germany; ^16^Research Center Neurosensory Science, Carl von Ossietzky University Oldenburg, Oldenburg, Germany; ^17^Department of Psychiatry and Psychotherapy III, University of Ulm, Ulm, Germany

**Keywords:** monkeypox, COVID-19, anxiety, vaccination, multi-national cross-sectional study

## Abstract

**Background:**

The aim of this study was to explore potential healthcare workers' (HCWs) concerns about the monkeypox virus in order to create practical solutions to manage this disease.

**Methods:**

Online cross-sectional research was conducted in 11 Arabic countries (Egypt, Saudi Arabia, Yemen, Syria, Libya, Algeria, Tunisia, Iraq, Palestine, Jordan, and Sudan) from 2 August 2022 to 28 December 2022.

**Results:**

Approximately 82% of respondents felt the need to acquire further information. The acceptability of the vaccine against monkeypox has been indicated by more than half of the participants (54.5%). Furthermore, we state that 45% of the participants are knowledgeable about the monkeypox virus, and 53.1% of the participants have never been affected with COVID-19 before are more worried about COVID-19 than about monkeypox. Participants diagnosed with COVID-19 were 0.63 times less likely to worry about monkeypox than those who were not diagnosed with COVID-19. A greater willingness to get the monkeypox vaccination was seen among the age group 21–30 years (42.4%) compared to the other age groups.

**Conclusion:**

Most healthcare professionals have a moderate knowledge of the monkeypox virus. Furthermore, they demonstrated a low willingness to get the vaccination against the monkeypox virus.

## 1. Introduction

Health experts are worried about the emergence of a new epidemic caused by the monkeypox virus, and they believe monkeypox virus may pose a new threat to human health when the world seems to be in the late stages of the coronavirus disease 2019 (COVID-19) pandemic (1, 2). Monkeypox virus is a DNA virus with two strands that belong to the genus *Orthopoxvirus*, which also contains variola, cowpox (CPX), and vaccinia viruses (3, 4). Since the Democratic Republic of the Congo (DRC) reported the first human cases of monkeypox in 1970, the disease has spread to other parts of Africa and, more recently, instances of spread of monkeypox outside of Africa have been reported (5). According to the World Health Organization (WHO), there have been more than 13,069 instances of monkeypox worldwide, as on 18 July 2022, with 80% of these cases occurring in the European Union (6). Sexual transmission of infections or diseases has been identified as a major factor associated with greater spreading of the current epidemic, particularly among males who have been identified as homosexual or bisexual (7). Moreover, the virus may get spread by sharing beds or clothes and through direct exposure to infected sores, scabs, or bodily fluids. While the symptoms of monkeypox are comparable with those of smallpox, the lesser extent of severity of the symptoms of monkeypox such as fever, rash, and lymphadenopathy characterize the clinical condition (8, 9). On the contrary, it is characterized by many complications, the most important of which are secondary bacterial infections, keratitis that threatens vision, encephalitis, and pneumonitis. As of late May 2022, many cases of monkeypox have been discovered in various countries in the Middle East (10).

As a result of the extraordinary success achieved by the World Health Organization (WHO) in smallpox eradication 40 years ago, smallpox vaccination is no longer used, with about 70% of the population worldwide have not been vaccinated. The smallpox vaccine is delineated as a prevention method for the monkeypox virus as well since it is effective against orthopoxvirus infections; however, most cases of monkeypox infection have occurred in non-vaccinated individuals (11). Healthcare workers are at high risk of contracting infectious diseases like the monkeypox virus. That increased risk stems from close contact between infected patients and healthcare staff, especially when personal protective equipment is unavailable. The third generation of the smallpox vaccine has shown high efficacy in healthcare workers (12): however, healthcare workers may decline vaccination because of emotional and personal considerations rather than scientific knowledge of this particular situation, and if they are affected by vaccine hesitancy, they may convey this attitude to the patients they care for (13). Healthcare workers must deal with the growing number of human monkeypox virus cases worldwide through early detection, management, and prevention. According to the WHO statement, one of the reasons for the resurgence of the infection was poor knowledge of monkeypox among healthcare workers (10). Before the monkeypox virus spreads further, it is necessary to renovate healthcare facilities and prepare for future epidemics, particularly in low-income countries with limited healthcare system resources (14). During the current COVID-19 pandemic, low- and middle-income courtiers have more reasons to worry about monkeypox virus due to their lower socioeconomic level and limited access to healthcare.

Consequently, they must prepare to cope with another outbreak (15). In Syria, the outbreak of COVID-19 has been a major challenge added to the country's inhabitants who were also affected by the catastrophic effects of warfare (16). A previous study from Jordan revealed that healthcare workers had limited knowledge of the monkeypox virus and confirmed that practitioners lacked confidence in their abilities to diagnose and treat infected patients (17). The monkeypox virus has been a source of rising worry among scientists for various circumstances, including the fact that the disease does not have a definitive treatment or vaccine until now, and the current treatment management depends on improving symptoms and preventing complications. Furthermore, after the monkeypox outbreak in many countries, concerns about the possibility of virus phenotype changing by different mutations have increased (18). The objective of this study is to assess the concerns of healthcare workers in the Arabic countries about the monkeypox virus and the factors associated with good knowledge, in addition to examining vaccine advocacy among them.

## 2. Methods

### 2.1. Study design and setting

An online cross-sectional study was conducted from 2 August 2022 to 28 December 2022 to assess worries and concerns among HCWs toward the monkeypox virus and the factors associated with good knowledge, as well as to examine monkeypox virus vaccine advocacy among them. The inclusion criteria were healthcare workers, such as physicians, nurses, pharmacists, and undergraduate medical students, from the Arabic countries. The countries involved in this study were Egypt, Saudi Arabia, Yemen, Syria, Libya, Algeria, Tunisia, Iraq, Palestine, Jordan, and Sudan. All participants were informed of the aim of the study, the work team identity, their right to withdraw from the study, and the confidentiality of their personal information. The questionnaire was developed based on a previous cross-sectional study conducted in the Arabic country, Saudi Arabia, which included validated scales (19). Furthermore, a professional translator translated the survey from English into Arabic to ensure the total comprehension of the questions. We performed convenience and snowball sampling strategies to perform a professional and non-biased data collection process as possible. We collected the data by creating a Google Forms survey and sending it to respondents through social media platforms such as Facebook, WhatsApp, and Telegram. Fourteen collaborators from each investigated Arabic country in our study were responsible for the data-gathering process. In addition, there was a lead collaborator in each involved study as a local investigator to monitor the data collection and investigate if there were any random, multi-auto, or illogical responses on the online questions, and to check the current job of each respondent to avoid including any person from non-medical staff.

### 2.2. Sample size calculation

The minimal sample size was computed by interrupting a single proportion of the population formula [*n* = [(*Z*α/2)2 × *P*(1 – *P*)]/*d*^2^], with a 95% of confidence level (CI); *Z*α/2 = 1.96; a 5% margin of error; *P*, the proportion of healthcare workers who were more concerned about Monkeypox disease compared to COVID-19 (35.7%); and the proportion of healthcare workers who accepted the vaccination (67.7%) (19). According to the formula, a sample size of 385 was required. The study questionnaire was sent to 3,902 participants through the Google Forms; however, 46 of them refused to participate, bringing the total number of responses to 3,856.

### 2.3. Measures

The questionnaire consists of 44 questions divided into five sections. The first section contains information about the participants' sociodemographic variables; the second evaluates HCWs' knowledge of the monkeypox virus and their sources of information; the third examines the perceptions and concerns of healthcare workers about the monkeypox virus; the fourth addresses questions regarding knowledge of HCWs monkeypox infection; and the final section of the questionnaire includes questions adapted from the Generalized Anxiety Disorder-7 (GAD-7) to assess HCWs anxiety about the monkeypox virus.

#### 2.3.1. Sociodemographic variables and professional characteristics

To identify about the participants' demographic characteristics, such as their age, country of origin, gender, marital status, place of residence, chronic disease, number of family members, economic status, and educational background (including whether they are physicians, nurses, pharmacists, or medical students and their academic year), 14 questions were included in the questionnaire. Furthermore, there were questions about the participants' working hospital type (primary, secondary, or tertiary healthcare centers). The respondents' years of experience and their workplace within the hospital if they work in the hospital pharmacy, intensive care units, isolation departments, or elsewhere were included as additional information. The last question of this section asked the respondents if they had ever been diagnosed with COVID-19.

#### 2.3.2. Healthcare workers' awareness and sources of information about monkeypox disease

This section consists of four questions about participants' awareness of the monkeypox virus, including whether the respondents had visited a monkeypox-endemic country (West or Central Africa, Europe, North America, the UAE, and Australia). Also, participants were asked to evaluate their current awareness of the monkeypox disease (low, high, or moderate), and they were asked how informed they were about monkeypox disease (international health websites, social media platforms, or scientific journals) and whether they needed to read more about monkeypox after participating in the survey.

#### 2.3.3. Perceptions and worries of healthcare workers about monkeypox disease

This section contains eight questions designed to measure the concerns and perceptions of healthcare workers regarding the monkeypox virus. Respondents were asked if they were concerned on whether the monkeypox virus will cause a global pandemic like COVID-19 and whether they believe that the monkeypox infection causes a more severe disease than monkeypox. In addition, they were asked to identify the cause of their monkeypox worries (such as their fear of being affected by the disease, concerns about developing another worldwide pandemic, or worries about national lockdown). Respondents were questioned on their acceptance of vaccination and their perceptions of which category should first get the monkeypox vaccine (older adult, children, college students, etc.).

#### 2.3.4. Knowledge of the monkeypox virus among healthcare workers

Regarding assessing HCWs' knowledge of the monkeypox virus, we adopted questionnaire items from a study about knowledge of human monkeypox among students in various Jordanian health schools (20). In this section, with 11 questions about monkeypox, participants were asked: “is monkeypox common in the Middle East?” “is monkeypox common in Western and Central Africa?” “is there a global epidemic of human monkeypox?” “is monkeypox caused by a virus or another pathogen?” and “is spreading the disease from person to person a risk?” Participants were also asked “whether human monkeypox could be treated with antibiotics?” “whether diarrhea is one of the signs or symptoms of human monkeypox?” “whether pustules are one of the signs or symptoms of infection?” “whether skin rash is one of the signs or symptoms of human monkeypox?” “whether monkeypox has similar signs and symptoms to smallpox?” and “whether vaccination is available to prevent human monkeypox?” The possible answers to each knowledge item were “yes,” “no,” and “I do not know”). Correct replies were given a score of 1, wrong responses were assigned a score of −1, and “I do not know” was given a score of 0. These scores represented the participants' monkeypox knowledge score (MPX K-score). An adequate degree of knowledge was determined as a score of 70% correct replies or above as we depended on the published studies.

#### 2.3.5. Generalized anxiety disorder toward monkeypox

This scale contains seven items that measure participants' GAD regarding the monkeypox virus (20, 21). Participants were asked to rate how often they had felt symptoms such as worry, concern, restlessness, impatience, and dread over the past 2 weeks. We assigned values from 0 to 3 for the four frequency levels of never, sometimes, often, and very frequently. There were four levels of severity determined by the GAD7 score: minimum (1–4), mild (5–9), moderate (10–14), and severe (0–14). (15–21).

### 2.4. Pilot study

To make sure the survey questions were clear before launching the online survey on social media platforms, we sent the questions to 45 randomly selected Arabic healthcare providers from specific countries. Then we modified the survey depending on the feedback and suggested adjustments. Although we have used the scales from a published study of an Arabic country, we ran a pilot study in which we sent the questionnaire to 50 volunteers, who were healthcare providers from those countries involved in our study to confirm the reliability of the used scales, for which we determined the Cronbach's α to each involved scale. Then, we confirmed that the scales we used in our cross-sectional study had high internal consistency levels (Cronbach's α was above 7.0).

### 2.5. Ethical consideration

The Syrian Ethical Society for Scientific Research at Aleppo University, Syria provided the ethical approval for conducting the study (IRB: SA-1087). In addition, we ordered at least one printed ethical approval from the clinical and educational institutions (Hospitals and Medical Colleges) of the lead collaborators from each investigated country of our study. The first question in the online survey was about the respondent's acceptance to complete the survey. We also ensured that all methods in our online cross-sectional were according to the Declaration of Helsinki developed by World Medical Association (WMA). The survey takes 5–12 min to complete, and for security purposes, all data is saved in an online database.

### 2.6. Statistical analysis

The data were examined using the Statistical Package for the Social Sciences (IBM SPSS V. 28.0). Statistical significance was defined as a *p*-value of ≤ 0.05. The quantitative data were given with a mean and standard deviation, while the categorical data were presented with frequency and percentages. After validating the data and distribution that were non-parametric using the Shapiro–Wilk test, we used the Kruskal–Wallis test to compare how much each subgroup differed from others in terms of their awareness of monkeypox, desiring to vaccinate themselves against monkeypox, and worrying toward the new pandemic that will arise due to monkeypox. Finally, using the cutoff points from the Saudi Arabian research (22), we conducted a binary logistic regression to calculate the odds ratios (ORs) between the dependent variables (awareness of monkeypox and desire to vaccinate themselves against monkeypox) and independent variables (sociodemographic factors) for having an appropriate awareness of monkeypox and a desire to vaccinate themselves against monkeypox.

## 3. Results

### 3.1. Demographic characteristics

The questionnaire was distributed to 3,902 participants; however, 46 among the participants declined to participate, resulting in a final sample size of 3,856. Most of the participants were aged between 21 and 30 years (78%), and more than half of the sampling participants (56.3%) were of females. Participants residing in the city comprised 82.3%, and most of the participants (50.2%) had a moderate financial condition. Students involved in the study were 50.1%, while practitioners involved in the study remained 30.7%. The majority (63.6%) of participants were employed by the hospital's central wards, while 16.5% worked in the outpatient department, and 12.1% were employed by the hospital's pharmacy or laboratory (Table 1).

**Table 1:** Participants' baseline sociodemographic and professional characteristics.

**Statement**		**Frequency**	**Percentage**
Country	Jordan	602	15.6%
	United Arab Emirates	14	0.36%
	Algeria	23	0.59%
	Saudi Arabia	264	6.8%
	Sudan	555	14.4%
	Somalia	9	0.2%
	Iraq	93	2.4%
	Kuwait	10	0.3%
	Morocco	8	0.2%
	Yemen	1,041	27.0%
	Tunisia	56	1.5%
	Oman	3	0.1%
	Syria	351	9.1%
	Palestine	40	1.0%
	Qatar	10	0.3%
	Lebanon	6	0.25%
	Libya	79	2.0%
Egypt	692	17.9%
Sex	Female	2,171	56.3%
	Male	1,685	43.7%
Age (years)	< 20	451	11.7%
	21–30	3,006	78.0%
	31–40	260	6.7%
	41–50	102	2.6%
	51–60	26	0.7%
	>60	9	0.2%
Marital state Households (family) size	Never married	3,107	80.6%
	Married	749	19.4%
	1–3 members	466	12.1%
	4–6 persons	1,873	48.6%
	7–10 persons	1,299	33.7%
	More than 10 persons	218	5.7%
Households' monthly income	Bad	248	6.4%
	Moderate	1,937	50.2%
	Good	1,341	34.8%
	Excellent	330	8.6%
Working hospital type	Primary healthcare center	1,569	40.7%
	Secondary healthcare hospital	1,134	29.4%
	Tertiary healthcare hospital	1,153	29.9%
Clinical role	Medical student	1,932	50.1%
	Technicians/lab workers and pharmacists	404	10.5%
	Nurses	337	8.7%
	Physicians	1,183	30.7%
Study year	First year	100	4.6%
	Second year	224	10.2%
	Third year	339	15.4%
	Fourth year	445	20.3%
	Fifth year	554	25.2%
	Sixth year	533	24.3%
Experience duration	< 5 years	2,054	84.7%
	More than 5 years	372	15.3%
Living place	Village	684	17.7%
	City	3,172	82.3%
Chromic disease	Don't have	3,559	92.3%
	Have	297	7.7%
Hospital working area/covering service	Pharmacy and laboratory	468	12.1%
	Critical care units	221	5.7%
	Infectious disease/isolation wards	81	2.1%
	General wards	2,451	63.6%
	OPD	635	16.5%

OPD, outpatient department.

### 3.2. HCWs' monkeypox disease perceptions and COVID-19 status

Participants with previous diagnoses of COVID-19 comprised 35.7%; however, 8.8% of participants were concerned that monkeypox might generate an epidemic like COVID-19, whereas 43.5% of participants were uncertain about the severity of monkeypox compared to smallpox. Respondents concerned more about monkeypox than about COVID-19 were 18.1%, and 82.3% of the respondents felt that they needed to learn more about it after reading the survey. More than half of the participants (54.5%) have expressed acceptance of the vaccination against monkeypox. Participants reported social media (58.1%), websites of the WHO/Centers for Disease Control and Prevention (CDC) (31.1%), and the Internet (30.2%) as a source of information about monkeypox (Table 2).

**Table 2:** Descriptive analysis of the HCWs' monkeypox disease perceptions and COVID-19 status.

**Statement**		**Frequency**	**Percentage**
Have you been previously diagnosed with COVID-19?	Yes	1,375	35.7%
	No	2,481	64.3%
Have you traveled in the last month to a country where monkeypox was recently reported?	I don't travel	3,665	95.1%
	Europe, North America, and Australia	54	1.4%
	UAE	63	1.6%
	West or Central Africa	24	0.6%
	Other (far Asia, India, Spain, France, and countries of Middle East)	50	1.3%
How would you rate your awareness of Monkeypox in the meantime?	Low	2,019	52.4%
	Moderate	1,656	42.9%
	High	181	4.7%
How worried are you that monkeypox can cause a worldwide pandemic like COVID-19?	None/less worried	1,991	51.6%
	Moderate worry	1,526	39.6%
	Worried a lot	339	8.8%
Do you think Monkeypox causes a more severe disease compared to Smallpox?	Disagree	666	17.3%
	Unsure	1,679	43.5%
	Agree	1,511	39.2%
Which is more worrisome to you, COVID-19 or monkeypox disease?	Unsure/equally worried	1,492	38.7%
	I am more worried about COVID-19	1,665	43.2%
	I am more worried about monkeypox	699	18.1%
Healthcare workers should apply more infection control measures than the current ones, with the new monkeypox outbreaks	Agree	3,069	79.6%
	Neither agree nor disagree	536	13.9%
	Disagree	251	6.5%
Please rate your worry level about traveling abroad with the new monkeypox outbreaks in some countries	Not worried at all	1,375	35.7%
	Somewhat worried	2,124	55%
	Extremely worried	357	9.3%
After Receiving this survey, did you perceive the need to read more about monkeypox disease?	No	682	17.7%
	Yes	3,174	82.3%
Your sources of information about monkeypox disease	Official local statements	1,163	30.1%
	International health authorities' websites (WHO or CDC)	1,202	31.1%
	Social media	2,244	58.1%
	Scientific journals	652	16.9%
	Other Internet-based sources	1,166	30.2%
Do you want to receive the monkeypox vaccine?	No	1,754	45.5%
	Yes	2,102	54.5%

### 3.3. HCWs' sources of worries from monkeypox disease

We found that 61.7% of participants were concerned about being infected themselves or their family, while 54.6% were worried about the number of monkeypox cases increasing to the level that might force a national lockdown. Less than half of the participants reported being anxious about the sickness progressing to the level of a global pandemic (45.9%) (Table 3).

**Table 3:** HCWs' sources of worries from monkeypox disease.

**Statement**	**Frequency**	**Percent**
Worried monkeypox might surge to cause national lockdown	2,107	54.6%
Me or my family being affected by the monkeypox	2,383	61.7%
Another worldwide pandemic	1,774	45.9%
International flight suspension	515	13.3%
Other	399	10.3%

### 3.4. The level of human monkeypox knowledge among HCW

**Figure 1:**
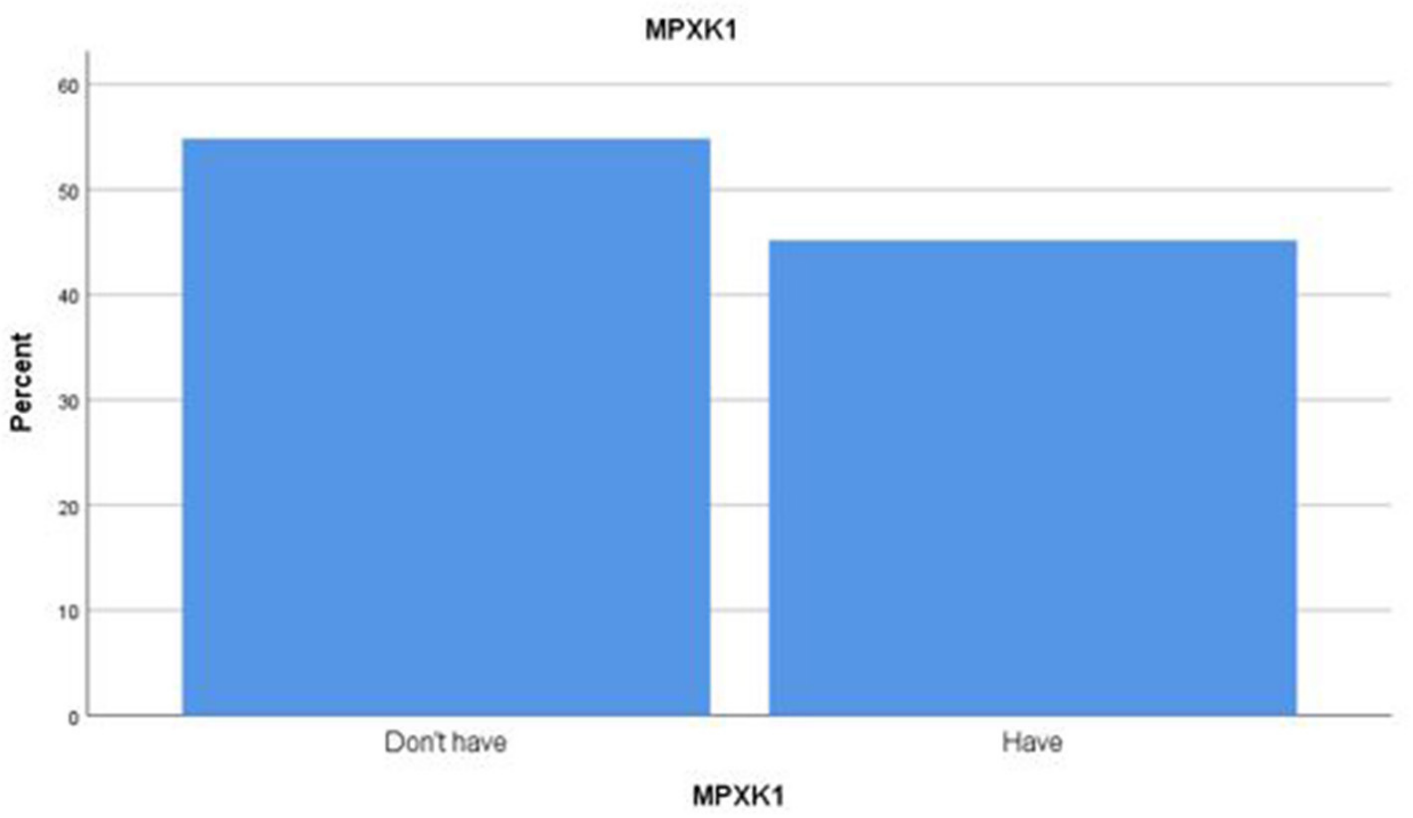
The prevalence of human monkeypox knowledge among HCW.

Approximately half of the participants (55%) were unaware of the monkeypox virus (Figure 1), and 23.8% of respondents believe that the monkeypox virus is expected in the Arabic countries. In comparison, 35.3% of respondents do not know whether there is a global epidemic of monkeypox. Regarding the resemblance of symptoms between monkeypox and smallpox, 58.4% of participants thought the symptoms were similar, while 23.3% of participants agreed that antibiotics might be used to treat monkeypox. Only 27.1% of respondents believe that monkeypox immunization is available (Table 4).

**Table 4:** The level of human monkeypox knowledge among HCWs.

**Human monkeypox knowledge item**	**Response**	**Frequency**	**Percent**
Monkeypox is prevalent in the Arabic countries	Incorrect	970	25.2%
	Do not know	1,967	51.0%
	Correct	919	23.8%
Monkeypox is prevalent in Southeast Asia	Incorrect	368	9.5%
	Do not know	2,201	57.1%
	Correct	1,287	33.4%
There is an outbreak of human monkeypox in the world	Incorrect	824	21.4%
	Do not know	1,363	35.3%
	Correct	1,669	43.3%
Monkeypox is caused by a virus	Incorrect	175	4.5%
	Do not know	908	23.5%
	Correct	2,773	71.9%
Human-to-human transmission of monkeypox occurs easily	Incorrect	778	20.2%
	Do not know	1,346	34.9%
	Correct	1,732	44.9%
Monkeypox and smallpox have similar signs and symptoms	Incorrect	284	7.4%
	Do not know	1,319	34.2%
	Correct	2,253	58.4%
Skin rash is one of the signs or symptoms of human monkeypox	Incorrect	193	5.0%
	Do not know	917	23.8%
	Correct	2,746	71.2%
Pustule is one of the signs or symptoms of human monkeypox	Incorrect	252	6.5%
	Do not know	1,630	42.3%
	Correct	1,974	51.2%
Antibiotics are used to treat human monkeypox	Incorrect	1,362	35.3%
	Do not know	1,595	41.4%
	Correct	899	23.3%
Diarrhea is one of the signs or symptoms of human monkeypox	Incorrect	554	14.4%
	Do not know	2,310	59.9%
	Correct	992	25.7%
Vaccination is available to prevent human monkeypox	Incorrect	876	22.7%
	Do not know	1,936	50.2%
	Correct	1,044	27.1%

### 3.5. HCWs' odds ratios of high worry of monkeypox compared to COVID-19

Our results show that females were more concerned about COVID-19 (44.6%) than monkeypox (11.7%), as well as participants who had not been diagnosed with COVID-19 were concerned more about COVID-19 (53.1%) than about monkeypox (11.2%). Among respondents who felt that monkeypox symptoms are like smallpox, 10.8% were more concerned about monkeypox than about COVID-19. Among anxious individuals, 18.4% are more concerned about COVID-19 than about monkeypox virus. Notably 7 out of 15 predictor factors were significantly linked with greater worry from monkeypox than from COVID-19 (*p* < 0.05). Participants with more than 5 years of work experience were less likely to be concerned more about monkeypox than about COVID-19 (OR = 0.59; 95% CI: 0.374–0.931), comparable to those with < 5 years of work experience. Participants diagnosed with COVID-19 were less likely to worry about monkeypox (OR = 0.63; 95% CI: 500–807) than those without. A higher likelihood of worrying about monkeypox than COVID-19 was anticipated among participants who worried more about monkeypox causing a widespread epidemic like COVID-19 (OR = 2.87; 95% CI:

1.962–4.212). Concern about monkeypox was expected to be higher than COVID-19 (OR = 4.47; 95% CI: 2.852–7.020) among participants who believed that monkeypox produces more severe symptoms than smallpox (Table 5).

**Table 5:** Multivariate binary logistic regression analysis of the HCWs' odds ratios of high worry from monkeypox compared to COVID-19.

**Variables**	**Categories**	**A high worry from monkeypox compared to COVID-19**				**P-value**	**Non-adjusted odds ratio (non-AOR)**	**Lower**	**Upper**	**P-value**	**Multivariate adjusted odds ratio (AOR)**	**Lower**	**Upper**
		**Worry more about COVID-19 or equal worrying**		**Worry from monkeypox**									
		**Frequency**	**Percentage**	**Frequency**	**Percentage**								
Age (years)	< 20	348	9.0%	103	2.7%	1							
	21–30	2,453	63.6%	553	14.3%	0.025	0.762	0.600	0.967	0.190	1.309	0.876	1.956
	31–40	226	5.9%	34	0.9%	0.002	0.508	0.333	0.775	0.547	1.225	0.633	2.370
	41–50	98	2.5%	4	0.1%	0.000	0.138	0.050	0.384	0.236	0.475	0.138	1.629
	51–60	24	0.6%	2	0.1%	0.089	0.282	0.065	1.211	0.967	0.965	0.184	5.074
	>60	7	0.2%	2	0.1%	0.965	0.965	0.197	4.718	0.351	2.266	0.406	12.639
Sex	Female	1,720	44.6%	451	11.7%	1							
	Male	1,437	37.3%	248	6.4%	0.000	0.658	0.555	0.780	0.216	0.859	0.676	1.093
Marital state	Not married	2,515	65.2%	592	15.4%	1							
	Married	642	16.6%	107	2.8%	0.000	0.658	0.555	0.780	0.781	1.049	0.748	1.472
Households (family) size	1–3 members	392	10.2%	74	1.9%	1							
	4–6 persons	1,551	40.2%	322	8.4%	0.499	1.100	0.835	1.449	0.277	1.235	0.844	1.809
	7–10 persons	1,036	26.9%	263	6.8%	0.040	1.345	1.013	1.784	0.024	1.581	1.063	2.351
	More than 10 persons	178	4.6%	40	1.0%	0.420	1.190	0.779	1.818	0.571	1.174	0.674	2.043
Clinical role	Medical student	1,566	40.6%	366	9.5%	1							
	Technicians/lab workers and pharmacists	323	8.4%	81	2.1%	0.608	1.073	0.820	1.404	0.234	1.276	0.854	1.906
	Nurses	282	7.3%	55	1.4%	0.253	0.834	0.612	1.138	0.674	0.897	0.540	1.489
	Physicians	986	25.6%	197	5.1%	0.107	0.855	0.706	1.034	0.411	1.120	0.855	1.468
Experience duration	< 5 years	1,669	68.8%	385	15.9%	1							
	More than 5 years	333	13.7%	39	1.6%	0.000	0.508	0.358	0.720	0.024	0.590	0.374	0.931
Chromic disease	Don't have	2,916	75.6%	643	16.7%	1							
	Have	241	6.3%	56	1.5%	0.735	1.054	0.778	1.427	0.240	1.272	0.852	1.898
Have you been previously diagnosed with COVID-19?	Yes	1,109	28.8%	266	6.9%								
	No	2,048	53.1%	433	11.2%	0.144	0.881	0.744	1.044	0.000	0.635	0.500	0.807
Have you traveled in the last month to a country where monkeypox was recently reported?	I didn't travel	2,995	77.7%	670	17.4%	1							
	Europe, North America, and Australia	43	1.1%	11	0.3%	0.694	1.144	0.587	2.229	0.480	0.692	0.249	1.922
	UAE	56	1.5%	7	0.2%	0.149	0.559	0.254	1.231	0.110	0.437	0.158	1.207
	West or Central Africa	21	0.5%	3	0.1%	0.469	0.639	0.190	2.147	0.672	0.752	0.201	2.813
	Other (far Asia, India, Spain, France, and middle eastern countries)	42	1.1%	8	0.2%	0.679	0.851	0.398	1.822	0.537	0.705	0.232	2.139
How would you rate your awareness of Monkeypox at the meantime?	Low	1,641	42.6%	378	9.8%	1							
	Moderate	1,374	35.6%	282	7.3%	0.183	0.891	0.752	1.056	0.024	0.757	0.595	0.963
	High	142	3.7%	39	1.0%	0.353	1.192	0.822	1.729	0.720	1.100	0.653	1.853
How worried are you that monkeypox can cause a worldwide pandemic similar to COVID-19?	Unsure/equally worried	1,778	46.1%	213	5.5%	1							
	I am more worried about COVID-19	1,173	30.4%	353	9.2%	0.000	2.512	2.087	3.024	0.000	1.706	1.311	2.221
	I am more worried about monkeypox	206	5.3%	133	3.4%	0.000	5.389	4.154	6.991	0.000	2.875	1.962	4.212
Do you think Monkeypox causes a more severe disease compared to smallpox?	Disagree	627	16.3%	39	1.0%	1							
	Unsure	1,436	37.2%	243	6.3%	0.000	2.721	1.915	3.864	0.002	2.094	1.322	3.318
	Agree	1,094	28.4%	417	10.8%	0.000	6.128	4.351	8.632	0.000	4.475	2.852	7.020
Healthcare workers should apply more infection control measures than the current ones, with the new Monkeypox outbreaks	Agree	2,444	63.4%	625	16.2%	1							
	Neither agree nor disagree	485	12.6%	51	1.3%	0.000	0.411	0.304	0.556	0.016	0.576	0.367	0.903
	Disagree	228	5.9%	23	0.6%	0.000	0.394	0.255	0.611	0.198	0.689	0.391	1.215
Please rate your worry level about traveling abroad with the new monkeypox outbreaks in some countries	Not worried at all	1,215	31.5%	160	4.1%	1							
	Somewhat worried	1,705	44.2%	419	10.9%	0.000	1.866	1.533	2.271	0.097	1.270	0.957	1.686
	Extremely worried	237	6.1%	120	3.1%	0.000	3.845	2.922	5.060	0.007	1.753	1.169	2.628
GAD-7	Don't have	2,448	63.5%	432	11.2%	1							
	Have anxiety	709	18.4%	267	6.9%	0.000	2.134	1.793	2.540	0.004	1.452	1.130	1.865

The logistic regression model was statistically significant, X2(31) = 284.591, *p* = 0.000. Hosmer and Lemeshow test 5.712 (*p* = 0.679). The model explained 18.3% (Nagelkerke *R*^2^) of factors associated with high worry from monkeypox compared to COVID-19.

### 3.6. HCWs' odds ratios of supporting vaccinations against monkeypox disease

Our analysis shows that 29.4% of females, 42.4% of participants aged between 21 and 30 years, 32.9% of participants diagnosed with COVID-19, and 46.6% of participants with < 5 years of experience accepted receiving the vaccine. However, 13.7% of participants with good economic status, 3.6% of participants aged between 31 and 40 years, and 35.3% of participants who were not anxious about monkeypox refused to receive the vaccine.

Notably, 7 of the 15 predictor factors were statistically associated with HCWs' support for immunizations against monkeypox (*p* < 0.05). Male MHWs were more likely to accept immunization against monkeypox (OR = 1.3; 95% CI: 1.168–1.668) than female MHWs. Participants aged between 21 and 30 years were 2.36 times more likely to receive the vaccine than those aged under 20. Participants who were not diagnosed with COVID-19 infection have a lower probability of accepting the vaccine than participants who were diagnosed with COVID-19 infection (OR = 0.64). Regarding the GAD-7 scale, anxious participants were more likely to endorse immunizations against monkeypox (OR = 1.48) than those without anxiety (Table 6).

**Table 6:** Multivariate binary logistic regression analysis of the HCWs' odds ratios of supporting vaccinations against monkeypox disease.

		**Do you want to receive the monkeypox vaccine**				***p*-value**	**Non-adjusted odds ratio (OR)**	**Lower**	**Upper**	***p*-value**	**Multivariate adjusted Odds Ratio (OR)**	**95% C.I.for EXP(B)**	**Upper**
		**No**		**Yes**								**Lower**	
		**Frequency**	**Percentage**	**Frequency**	**Percentage**								
Sex	Female	1,038	26.9%	1,133	29.4%								
	Male	716	18.6%	969	25.1%	0.001	1.240	1.091	1.409	0.000	1.396	1.168	1.668
Ageo	< 20	189	4.9%	262	6.8%	0.008		0.004	
	21–30	1,372	35.6%	1,634	42.4%	0.137	0.859	0.703	1.050	0.173	0.807	0.593	1.098
	31–40	140	3.6%	120	3.1%	0.002	0.618	0.455	0.841	0.130	0.689	0.426	1.115
	41–50	34	0.9%	68	1.8%	0.112	1.443	0.918	2.268	0.021	2.362	1.136	4.911
	51–60	13	0.3%	13	0.3%	0.418	0.721	0.327	1.591	0.602	0.764	0.277	2.103
	>60	4	0.1%	5	0.1%	0.879	0.902	0.239	3.403	0.729	0.777	0.187	3.224
Marital state	Not married	1,409	36.5%	1,698	44.0%					
	Married	345	8.9%	404	10.5%	0.725	0.972	0.828	1.140	0.382	0.901	0.713	1.139
Working hospital type	Primary healthcare center	711	18.4%	858	22.3%	0.950				0.449			
	Secondary care hospital	514	13.3%	620	16.1%	0.996	1.000	0.857	1.165	0.962	1.005	0.816	1.238
	Tertiary care hospital	529	13.7%	624	16.2%	0.770	0.977	0.839	1.139	0.272	0.889	0.722	1.096
Experience duration	< 5 years	928	38.3%	1,126	46.4%								
	More than 5 years	163	6.7%	209	8.6%	0.627	1.057	0.846	1.320	0.522	0.901	0.655	1.240
Have you been previously diagnosed with COVID-19?	yes	541	14.0%	834	21.6%								
	No	1,213	31.5%	1,268	32.9%	0.000	0.678	0.593	0.775	0.000	0.642	0.534	0.773
Healthcare workers should apply more infection control measures than the current ones, with the new Monkeypox outbreaks	Agree	1,296	33.6%	1,773	46.0%	0.000				0.000			
	Neither agree nor disagree	293	7.6%	243	6.3%	0.000	0.606	0.504	0.729	0.001	0.627	0.479	0.820
	Disagree	165	4.3%	86	2.2%	0.000	0.381	0.291	0.499	0.000	0.408	0.288	0.579
Households' monthly income	Bad	130	3.4%	118	3.1%	0.000				0.003			
	Moderate	944	24.5%	993	25.8%	0.275	1.159	0.889	1.510	0.096	1.348	0.948	1.917
	Good	530	13.7%	811	21.0%	0.000	1.686	1.284	2.213	0.013	1.586	1.102	2.283
	Excellent	150	3.9%	180	4.7%	0.098	1.322	0.950	1.839	0.001	2.121	1.359	3.311
After receiving this survey, did you perceive the need to read more about monkeypox disease?	No	466	12.1%	216	5.6%								
	Yes	1,288	33.4%	1,886	48.9%	0.000	3.159	2.649	3.768	0.000	3.068	2.427	3.877
Gad7	Don't have	1,360	35.3%	1,520	39.4%								
	Have anxiety	394	10.2%	582	15.1%	0.000	1.322	1.141	1.531	0.000	1.482	1.222	1.797
Your sources of Information about monkeypox disease	Official local statements	112	2.9%	116	3.0%	0.000				0.073			
	International health authorities' websites (WHO or CDC)	122	3.2%	154	4.0%	0.271	1.219	0.857	1.733	0.565	1.138	0.733	1.765
	Social media	602	15.6%	570	14.8%	0.536	0.914	0.688	1.214	0.169	0.777	0.543	1.113
	Scientific journals	38	1.0%	48	1.2%	0.435	1.220	0.741	2.008	0.958	1.017	0.545	1.897
	Other internet-based sources	207	5.4%	215	5.6%	0.986	1.003	0.727	1.384	0.687	0.919	0.609	1.386
	more than one source	673	17.5%	999	25.9%	0.011	1.433	1.086	1.891	0.788	1.049	0.739	1.489
Constant		0.043	0.537		

The logistic regression model was statistically significant, X2(23)= 254.087, *p* = 0.000. Hosmer and Lemeshow test 8.258 (*p* = 0.408). The model explained 13.3% (Nagelkerke *R*^2^) of factors associated with supporting vaccinations against monkeypox disease.

### 3.7. HCWs' odds ratios of supporting the implementation of tighter infection control measures against monkeypox compared to the currently applied during COVID-19

Regarding adherence to monkeypox disease control measures, participants who showed no adherence were 53.7% of females, 73.1% of participants aged between 21 and 30 years, 38.9% of participants worried more about COVID-19, and 23.6% of respondents had anxiety about monkeypox. In comparison, participants revealed adherence to control measures were 4.9% of participants aged between 31 and 40 years, 4.8% of participants who do not have anxiety about monkeypox, and 5.9% of participants with < 5 years of experience.

Notably, five of the 14 predictor factors were substantially linked to HCWs' probability of backing more stringent infection control measures against monkeypox (*p* < 0.05). Females have shown greater adherence to disease control measures than males (OR = 1.67). Participants with anxiety were more likely to adhere to disease control measures than that of the participants without anxiety (OR = 1.79). Participants worried more about COVID-19 have a greater probability of disease control measures adherence (OR = 1.64) compared to participants who expressed equal concern about both illnesses (Table 7).

**Table 7:** Multivariate binary logistic regression analysis of the HCWs' odds of supporting the implementation of tighter infection control measures against monkeypox compared to the currently applied during COVID-19.

**Variable**	**Subgroups**	**Tighter infection control measures**				**P-value**	**Non-adjusted OR**	**Lower**	**Upper**	**P-value**	**Multivariate adjusted OR**	**Lower**	**Upper**
		**Not doing**		**Doing tighter control measures**									
		**Frequency**	**Percentage**	**Frequency**	**Percentage**								
Sex	Female	2,071	53.7%	100	2.6%								
	Male	1,534	39.8%	151	3.9%	0.000	2.039	1.570	2.647	0.002	1.678	1.200	2.347
Age (years)	< 20	432	11.2%	19	0.5%	0.002				0.716			
	21–30	2,817	73.1%	189	4.9%	0.086	1.525	0.942	2.471	0.282	1.487	0.721	3.066
	31–40	229	5.9%	31	0.8%	0.000	3.078	1.701	5.570	0.318	1.659	0.614	4.479
	41–50	94	2.4%	8	0.2%	0.131	1.935	0.822	4.553	0.968	0.972	0.244	3.866
	51–60	22	0.6%	4	0.1%	0.017	4.134	1.296	13.190	0.591	0.516	0.046	5.749
	>60	9	0.2%	0	0.0%	0.999	0.000	0.000	.	0.999	0.000	0.000	.
Marital state	Not married	2,914	75.6%	193	5.0%								
	Married	691	17.9%	58	1.5%	0.128	1.267	0.934	1.719	0.448	0.824	0.499	1.359
Experience duration	< 5 years	1,912	78.8%	142	5.9%								
	More than 5 years	335	13.8%	37	1.5%	0.041	1.487	1.017	2.175	0.084	1.642	0.936	2.882
After receiving this survey, did you perceive the need to read more about Monkeypox disease?	No	570	14.8%	112	2.9%								
	Yes	3,035	78.7%	139	3.6%	0.000	0.233	0.179	0.304	0.000	0.360	0.253	0.513
GAD-7	Don't have	2,696	69.9%	184	4.8%								
	Have anxiety	909	23.6%	67	1.7%	0.603	1.080	0.808	1.443	0.003	1.791	1.218	2.633
Your sources of information about Monkeypox disease	Official local statements	203	5.3%	25	0.6%	0.000				0.031			
	International health authorities' websites (WHO or CDC)	242	6.3%	34	0.9%	0.638	1.141	0.659	1.975	0.077	1.883	0.933	3.798
	Social media	1,117	29.0%	55	1.4%	0.000	0.400	0.244	0.656	0.560	0.831	0.445	1.550
	Scientific journals	79	2.0%	7	0.2%	0.462	0.719	0.299	1.730	0.999	1.000	0.345	2.899
	Other Internet-based sources	392	10.2%	30	0.8%	0.094	0.621	0.356	1.085	0.835	0.925	0.446	1.922
	More than one source	1,572	40.8%	100	2.6%	0.005	0.517	0.325	0.820	0.352	0.753	0.415	1.367
Clinical role	Medical student	1,822	47.3%	110	2.9%	0.010				0.232			
	Technicians/lab workers and pharmacists	386	10.0%	18	0.5%	0.321	0.772	0.464	1.287	0.105	0.499	0.215	1.155
	Nurses	307	8.0%	30	0.8%	0.025	1.619	1.062	2.467	0.553	1.224	0.628	2.387
	Physicians	1,090	28.3%	93	2.4%	0.018	1.413	1.062	1.881	0.277	0.809	0.552	1.185
Chromic disease	No	3,334	86.5%	225	5.8%								
	Have	271	7.0%	26	0.7%	0.104	1.422	0.930	2.173	0.967	1.011	0.584	1.751
How worried are you that monkeypox can cause a worldwide pandemic similar to COVID-19?	None/less worried	1,833	47.5%	158	4.1%	0.000				0.862			
	Moderate worry	1,444	37.4%	82	2.1%	0.003	0.659	0.500	0.868	0.697	0.928	0.637	1.352
	Worried a lot	328	8.5%	11	0.3%	0.003	0.389	0.209	0.725	0.802	1.099	0.525	2.300
Please rate your worry level about traveling abroad with the new Monkeypox outbreaks in some countries	Not worried at all	1,241	32.2%	134	3.5%	0.000				0.303			
	Somewhat worried	2,020	52.4%	104	2.7%	0.000	0.477	0.366	0.622	0.143	0.761	0.528	1.097
	Extremely worried	344	8.9%	13	0.3%	0.000	0.350	0.196	0.626	0.344	0.685	0.313	1.499
Which is more worrisome to you, COVID-19 or monkeypox disease?	Unsure/equally worried	1,429	37.1%	63	1.6%	0.000				0.022			
	I am more worried about COVID-19	1,500	38.9%	165	4.3%	0.000	2.495	1.850	3.365	0.010	1.642	1.124	2.397
	I am more worried about monkeypox	676	17.5%	23	0.6%	0.296	0.772	0.475	1.255	0.947	1.021	0.551	1.891
Do you think monkeypox causes a more severe disease compared to smallpox?	Disagree	544	14.1%	122	3.2%	0.000				0.000			
	Unsure	1,599	41.5%	80	2.1%	0.000	0.223	0.166	0.301	0.000	0.315	0.215	0.463
	Agree	1,462	37.9%	49	1.3%	0.000	0.149	0.106	0.211	0.000	0.224	0.141	0.357
Households (family) size	1–3 members	435	11.3%	31	0.8%	0.880				0.995			
	4–6 persons	1,753	45.5%	120	3.1%	0.847	0.961	0.638	1.445	0.850	1.051	0.627	1.761
	7–10 persons	1,216	31.5%	83	2.2%	0.843	0.958	0.625	1.468	0.989	1.004	0.582	1.731
	more than 10 persons	201	5.2%	17	0.4%	0.585	1.187	0.642	2.194	0.929	1.035	0.485	2.211

The logistic regression model was statistically significant, X2(30) = 200.97, *p* = 0.000. Hosmer and Lemeshow test 3.89 (*p* = 0.866). The model explained 19.4% (Nagelkerke *R*^2^) of factors associated with the supporting implementation of tighter infection control measures against monkeypox compared to the currently applied during COVID-19.

### 3.8. HCWs' odds ratios of monkeypox knowledge score

Good knowledge about the monkeypox virus was shown by 25.4% of females and 34.8% of individuals aged 21–30 years. However, only 21.3% of medical students and 7.7% of clinicians with more than 5 years of experience show adequate knowledge of monkeypox. Only 22.2% of the participants agreed that monkeypox develops a more severe illness than smallpox, and 27.6% of respondents who agreed to receive the monkeypox vaccination had good knowledge of monkeypox. Only 12.1% of individuals with anxiety disorders have a good knowledge of monkeypox. In a multivariate logistic regression analysis, we found that family size, study year, participants' ratings of their awareness of monkeypox, participants' worry that monkeypox will cause a pandemic like COVID-19, and whether healthcare workers should apply more infection control measures were all significantly associated with HCWs' odds ratios of knowing about monkeypox (*p* < 0.05). Participants concerned about monkeypox developing a similar pandemic like COVID-19 have greater knowledge than participants who did not concern about monkeypox (OR = 1.82). Respondents who disagreed that HCWs should adhere more to the disease control methods were less likely to be knowledgeable about monkeypox than participants who agreed that HCWs should adhere more to the disease control methods (OR= 0.38) (Table 8).

**Table 8:** Multivariate binary logistic regression analysis of the HCWs' odds ratios of monkeypox knowledge score.

**Variable**	**Subgroups**	**Monkeypox knowledge**				***p*-value**	**Non-adjusted OR**	**Lower**	**Upper**	***p*-value**	**Multivariate adjusted OR**	**Lower**	**Upper**
		**Don't have**		**Have**									
		**Frequency**	**Percentage**	**Frequency**	**Percentage**								
Sex	Female	1,191	30.9%	980	25.4%	1							
	Male	923	23.9%	762	19.8%	0.959	1.003	0.883	1.140	0.127	0.807	0.613	1.063
Age	< 20	258	6.7%	193	5.0%	1							
	21–30	1,664	43.2%	1,342	34.8%	0.461	1.078	0.883	1.317	0.558	1.154	0.715	1.861
	31–40	142	3.7%	118	3.1%	0.502	1.111	0.817	1.510	0.755	1.219	0.351	4.233
	41–50	30	0.8%	72	1.9%	0.000	3.208	2.015	5.107	1.000	0.000	0.000	
	51–60	16	0.4%	10	0.3%	0.664	0.835	0.371	1.882	1.000	0.000	0.000	
	>60	3	0.1%	6	0.2%	0.168	2.674	0.660	10.825	0.999	0.000	0.000	
Marital state	Not married	1,764	45.7%	1,343	34.8%	1							
	Married	350	9.1%	399	10.3%	0.000	1.497	1.276	1.757	0.183	1.410	0.851	2.337
Households (family) size	1–3 members	260	6.7%	206	5.3%	1							
	4–6 persons	1,013	26.3%	860	22.3%	0.507	1.072	0.874	1.314	0.292	1.293	0.802	2.087
	7–10 persons	739	19.2%	560	14.5%	0.682	0.956	0.773	1.184	0.230	1.352	0.826	2.212
	More than 10 persons	102	2.6%	116	3.0%	0.028	1.435	1.039	1.982	0.011	2.220	1.197	4.118
Working hospital type	Primary healthcare center	861	22.3%	708	18.4%	1							
	Secondary care hospital	591	15.3%	543	14.1%	0.156	1.117	0.959	1.302	0.460	0.886	0.644	1.220
	Tertiary care hospital	662	17.2%	491	12.7%	0.187	0.902	0.774	1.051	0.527	0.896	0.638	1.259
Clinical role	Medical student	1,111	28.8%	821	21.3%	1							
	Technicians/lab workers and pharmacists	204	5.3%	200	5.2%	0.010	1.327	1.070	1.645	0.533	0.813	0.425	1.557
	Nurses	172	4.5%	165	4.3%	0.027	1.298	1.030	1.637	0.150	1.452	0.873	2.414
	Physicians	627	16.3%	556	14.4%	0.014	1.200	1.037	1.388	0.572	0.853	0.491	1.481
Study year	First year	56	2.6%	44	2.0%	1							
	Second year	119	5.4%	105	4.8%	0.632	1.123	0.699	1.804	0.770	1.110	0.550	2.240
	Third year	206	9.4%	133	6.1%	0.393	0.822	0.523	1.290	0.088	0.548	0.274	1.094
	Fourth year	256	11.7%	189	8.6%	0.780	0.940	0.607	1.455	0.140	0.572	0.272	1.202
	Fifth year	365	16.6%	189	8.6%	0.059	0.659	0.428	1.015	0.012	0.383	0.181	0.812
	Sixth Year	284	12.9%	249	11.3%	0.617	1.116	0.726	1.715	0.392	0.721	0.340	1.526
Experience duration	< 5 years	1,122	46.2%	932	38.4%	1							
	More than 5 years	185	7.6%	187	7.7%	0.082	1.217	0.976	1.518	0.121	0.645	0.371	1.122
Chromic disease	Don't have	1,940	50.3%	1,619	42.0%	1							
	Have	174	4.5%	123	3.2%	0.175	0.847	0.666	1.077	0.723	1.088	0.683	1.734
Hospital working area/covering service	Pharmacy and laboratory	253	6.6%	215	5.6%	1							
	Critical care units	129	3.3%	92	2.4%	0.288	0.839	0.607	1.160	0.369	0.698	0.318	1.530
	Infectious disease/isolation wards	32	0.8%	49	1.3%	0.016	1.802	1.114	2.915	0.688	1.291	0.371	4.489
	General wards	1,356	35.2%	1,095	28.4%	0.614	0.950	0.779	1.159	0.549	1.182	0.684	2.041
	OPD	344	8.9%	291	7.5%	0.970	0.995	0.783	1.265	0.651	0.863	0.457	1.632
Have you been previously diagnosed with COVID-19?	Yes	709	18.4%	666	17.3%	1							
	No	1,405	36.4%	1,076	27.9%	0.002	0.815	0.714	0.931	0.456	1.121	0.830	1.516
Have you traveled in the last month to a country where monkeypox was recently reported?	I didn't travel	1,988	51.6%	1,677	43.5%	1							
	Europe, North America, and Australia	37	1.0%	17	0.4%	0.039	0.545	0.306	0.971	0.062	0.213	0.042	1.082
	UAE	42	1.1%	21	0.5%	0.052	0.593	0.350	1.005	0.060	0.365	0.128	1.044
	West or Central Africa	19	0.5%	5	0.1%	0.021	0.312	0.116	0.837	0.435	0.558	0.129	2.414
	Other (far Asia, India, Spain, France, and countries from Middle East)	28	0.7%	22	0.6%	0.804	0.931	0.531	1.634	0.766	1.169	0.418	3.273
How would you rate your awareness of Monkeypox at the meantime?	Low	1,290	33.5%	729	18.9%	1							
	Moderate	722	18.7%	934	24.2%	0.000	2.289	2.004	2.615	0.000	1.828	1.390	2.405
	High	102	2.6%	79	2.0%	0.045	1.371	1.008	1.864	0.924	0.969	0.508	1.849
How worried are you that monkeypox can cause worldwide pandemic similar to COVID-19?	None/less worried	1,198	31.1%	793	20.6%	1							
	Moderate worry	749	19.4%	777	20.2%	0.000	1.567	1.370	1.793	0.003	1.577	1.171	2.124
	Worried a lot	167	4.3%	172	4.5%	0.000	1.556	1.235	1.960	0.047	1.627	1.006	2.632
Do you think monkeypox causes more severe disease compared to smallpox?	Disagree	399	10.3%	267	6.9%	1							
	Unsure	1,059	27.5%	620	16.1%	0.154	0.875	0.728	1.052	0.626	0.903	0.600	1.360
	Agree	656	17.0%	855	22.2%	0.000	1.948	1.618	2.344	0.299	1.254	0.818	1.921
Which is more worrisome to you, COVID-19 or monkeypox disease?	Unsure/equally worried	858	22.3%	634	16.4%	1							
	I am more worried about COVID-19	898	23.3%	767	19.9%	0.044	1.156	1.004	1.331	0.138	1.256	0.929	1.699
	I am more worried about monkeypox	358	9.3%	341	8.8%	0.006	1.289	1.076	1.544	0.509	1.132	0.783	1.636
Healthcare workers should apply more infection control measures than the current ones, with the new monkeypox outbreaks	Agree	1,605	41.6%	1,464	38.0%	1							
	Neither agree nor disagree	332	8.6%	204	5.3%	0.000	0.674	0.558	0.813	0.002	0.464	0.289	0.746
	Disagree	177	4.6%	74	1.9%	0.000	0.458	0.346	0.607	0.001	0.389	0.220	0.689
Please rate your worry level about traveling abroad with the new monkeypox outbreaks in some countries	Not worried at all	894	23.2%	481	12.5%	1							
	Somewhat worried	1,053	27.3%	1,071	27.8%	0.000	1.890	1.644	2.174	0.720	0.945	0.695	1.286
	Extremely worried	167	4.3%	190	4.9%	0.000	2.115	1.671	2.676	0.280	0.764	0.469	1.245
Do you want to receive Monkeypox vaccine	No	1,078	28.0%	676	17.5%	1							
	Yes	1,036	26.9%	1,066	27.6%	0.000	1.641	1.443	1.866	0.370	1.133	0.862	1.488
GAD-7	Don't have	1,605	41.6%	1,275	33.1%	1							
	Have anxiety	509	13.2%	467	12.1%	0.052	1.155	0.999	1.336	0.172	0.813	0.605	1.094

The logistic regression model was statistically significant, X2(45) = 147.186, *p* = 0.000. Hosmer and Lemeshow test 5.554 (*p* = 0.697). The model explained 16.8% (Nagelkerke *R*^2^) of factors associated with monkeypox knowledge score.

## 4. Discussion

Monkeypox is an infectious disease caused by orthopoxvirus characterized by a rash that may be isolated, preceded, or accompanied by fever or lymph nodes (23). Since 14 May 2022, confirmed cases of the virus have been reported or confirmed in several countries in Europe and North America, and the situation is evolving rapidly. In the UK, 16 cases of infection have been detected (as of 17 May 2022). Except for the first infected person, returning from Nigeria, all appear to have been infected in the UK, according to the local health safety agency (24). For fear of a possible new pandemic, health authorities worldwide have boosted their efforts to ensure the control of its spread by studying the means of transmission and early clinical signs. During the current increase of the reported infected cases of monkeypox, the knowledge, concern, and perception of the available vaccines are concerning factors, especially among healthcare providers and medical staff persons (25). Our study was conducted in the Middle East and North Africa (MENA) region with a final sample size of 3,856 participants, of which 1,375 had a history of COVID-19 diagnosis. Results reported that about 9% of participants considered that monkeypox might generate as an epidemic and a tremendous burden on human health scenario might occur like COVID-19, while 51.6% had no worries about monkeypox. These findings are similar to a Saudi Arabian study where only 25.3% of the study population were very worried, and 48.7% had no or less worries about a further monkeypox pandemic (22). Also, for almost the quarter, 18.1% of participants were concerned more about monkeypox than about COVID-19. These findings are also concomitant with another Saudi Arabian study published in August 2022 carried out by Mohamad et al. among the general population, where results reported a higher worry (62%) about COVID-19 than monkeypox (26). Concerning HCWs' sources of worries toward monkeypox disease, the majority (61.7%) of participants were concerned about being infected themselves or their relatives, and slightly more than half (54%) were afraid of a possible future lockdown. Similarly, these findings are concomitant with results found in a Saudi Arabian study by Temsah et al. (26).

About monkeypox knowledge level, slightly more than half (55%) of the participants were unaware of the monkeypox virus and had no sufficient information about it, and 58.4% of respondents could not make a difference between monkeypox and smallpox symptoms. Also, only 27.1% of participants reported positively that monkeypox immunization is already available. Knowledge findings were concomitant overall with Indonesian research conducted by Harapan et al., where monkeypox knowledge level was evaluated as low at 63.5% and insufficient among 432 general practitioners (27).

Male HCWs in the MENA region were less predicted to worry about monkeypox than female HCWs (6.4 and 11.7%, respectively), which was also reported toward COVID-19 worries. This was similarly found in a Saudi Arabian study by Ajman et al. (22). In addition, results reported that participants diagnosed with COVID-19 were less likely to worry about monkeypox (OR = 0.63 times) than those who had not been infected with COVID-19 virus. Findings also reported higher acceptance for the monkeypox vaccine by participants who had not been diagnosed with COVID-19 (*n* = 1,268, 32.9%) than those had been diagnosed with previous COVID-19. This incomprehensible and unpredictable finding could only be justified by a drop in healthcare workers' confidence level in vaccine protection after COVID-19 infection following administration of vaccines. This should be adjusted and corrected as approved vaccines have proven to be effective in preventing fatal complications of COVID-19 infection, reducing the number of people hospitalized and admitted to the intensive care unit, and reducing the number of infections without preventing it (28).

The knowledge of monkeypox infection, attitude toward its possible spread among healthcare practitioners in the MENA region, and vaccine advocacy must be improved urgently. This will prevent a possible pandemic because a good knowledge of the symptoms, confidence in diagnosis, modes of transmission, physiopathology, and comorbidities will help to avoid the maximum number of cases. Also, in case of a further pandemic, it will pave way to control the situation efficiently and professionally, based on the previous COVID-19 experience (15). These human monkeypox concerns among Arabic healthcare professionals can be corrected and improved through several approaches and by multiple means such as (29–32) (a) continuing medical education and scientific improvement on the infection process, which makes it less contagious than COVID-19 and, therefore, same rapid spread and a sudden pandemic scenario like COVID-19 are not expected; (b) more data about available vaccines and their efficiency—currently, only two vaccines, ACAM2000 vaccine and JYNNEOS, known as Imvanex, are available, and (c) involvement in research of international monkeypox network and patients' sensitivity and education on preventive measures.

## 5. Limitations and strengths

To examine the present degree of opinions of healthcare professionals concerning the characteristics of the monkeypox epidemic, next to COVID-19, our international cross-sectional survey includes a large sample size from various countries in the Arabic region. Additionally, we utilized scales that were getting better with its effectiveness, developed by Arabic scholars. We checked their validity to ensure if the questions they were using accurately represented the subject being investigated. Nevertheless, even though cross-sectional research may be carried out in a short amount of time and at no cost, the research needs to consider the specific and valid causal link and the generality of monkeypox. In addition, concerning the online cross-sectional research, it is difficult to get answers from those who do not have surplus time for attempting the questionnaire, those who do not have Internet access and a mobile phone, or those who are having trouble completing the survey due to technical challenges. This is particularly the case in connection with older adult individuals who are not familiar with the use of mobile phones.

## 6. Conclusion

Our results showed that healthcare professionals in the Arabic countries seemed to be less concerned about the monkeypox virus compared with their concern about the COVID-19 virus. Moderate knowledge of the monkeypox virus was noticed, and less tendency to receive vaccination against the monkeypox virus was also noticed. Furthermore, negative attitudes toward the monkeypox virus protection methods were observed. As a result, we recommend further regulations for the medical staff and precautionary measures. Furthermore, adequate awareness programs should be implemented for medical staff to teach them about the risks of monkeypox infection.

## Data availability statement

**T**he original contributions presented in the study are included in the article/Supplementary material, further inquiries can be directed to the corresponding authors.

## Ethics statement

The Syrian Ethical Society for Scientific Research at Aleppo University, Syria, provided the ethical approval for conducting the study (IRB: SA-1087). In addition we ordered at least one printed ethical approval from the lead collaborator from each inquired country in our study that was given by the clinical, and educational institutions (Hospitals, Medical Colleges). To confirm that participating in our study was voluntary, the first question in the online survey was about the respondent's acceptance to complete the survey.

## Author contributions

SS: study conception and design. HA, HB, NJ, MR, MN, WH, BS, AA, and SF: writing and reviewing the manuscript. MN and SS: formal analysis. IA, SA, and RY: draft manuscript. AR and ME: interpretation of results and revising. All authors reviewed the results and approved the final version of the manuscript.

## Data collection group

1. Albaraa Daradkeh, Faculty of Medicine, Alexandria University, Alexandria, Egypt (elbaraa.mahmoud1901@alexmed.edu.eg).
2. Zuhair Antakly, Resident Doctor at Aleppo University Hospital, Department of Oncology (Zuhairantakly@gmail.com).
3. Mirna Maged Armanyos, Faculty of Medicine, Alexandria University, Egypt (mirnam.armanios@icloud.com).
4. Ajmal J. Alsufyani, Faculty of Medicine, Sana'a University, Sana'a, Yemen (ajmalalsufyni@gmail.com).
5. Salsabeel Abdulbaki Ghalib Al_hanani: Faculty of Medicine, Sana'a University, Sana'a, Yemen (salsabeelalhanani@gmail.com).
6. Moyasar Awad Idris Abubakr, House Officer, Gadarif Teaching Hospital, Surgical Department Gadarif, Sudan (mayasarawad@gmail.com).
7. Hadeil Ali Abdelsalam Alhashmi, Faculty of Medicine, Altinbaş University, Istanbul, Turkey (Hadeilalhashmi@gmail.com).
8. Jarjees Abduljabar Sulaiman, University of Duhok, College of Medicine (jarjesabduljabar@gmail.com).
9. Hadeel Fuad Alwan Al-sharjabi, Faculty of Medicine, Sana'a University, Sana'a, Yemen (hadeelfuadalsharjabi89@gmail.com).
10. Nidaa T Alhumaidi, Faculty of Pharmacy, Taif University, Taif, Saudi Arabia (nidaa.t.alhumidi@icloud.com).
11. Monzir Ahmed Hassan Osman, Department of General Surgery, Ibrahim Malik teaching hospital, Khartoum, Sudan (monzirahmed97@gmail.com).
12. Mohamed Basyouni Helal, Faculty of Medicine, Menoufia University, Menoufia, Egypt (mohamedbasyouni416843@gmail.com).
13. Abdulghani A Al-Aswadi, Faculty of Medicine, Sana'a University, Sana'a, Yemen (abdulghanialaswadi1@gmail.com).
14. Amna Elaagib, MBBS, Almughtaribeen University, Khartoum, Sudan (amnaelaagib@gmail.com).
15. Amatalkhaleq Hussein Hamoud Azzam, University of Sana'a, Faculty of Medicine and Health Science, Sana'a, Yemen (amatalkhaleqazzam@gmail.com).
16. Alshaymaa Mortada Ali Eltohry, Faculty of Medicine, Ain Shams University, Egypt (alshaymaaali62@gmail.com).
17. Wesal Mohammed Ahmed Abdelkaream Ishag, Faculty of Medicine, University of Gezira (wesal92sola@gmail.com).
18. Lyn Ahmad, Faculty of Medicine, Damascus University, Damascus, Syria (lynros62@gmail.com).
19. Safiya Mohammed Al-Haddi, Thamar University, Faculty of Medicine, Yemen (Saffimohammed979@gmail.com).
20. Ebrahim Ahmed Shaddad, Faculty of Medicine, Sana'a University, Sana'a, Yemen (ebrahimshaddad.es@gmail.com).
21. Mawahib Hajhamed El Sheikh Mergani, Faculty of Medicine, Ahfad University for Women, Khartoum, Sudan (mawahibhajhamed@gmail.com).
22. Hossam Tharwat Ali, Qena Faculty of Medicine, South Valley University, Qena, Egypt (hossamtharwatali@gmail.com).
23. Hadeel Shayef Ahmed Aljalal, Faculty of Medicine, Sana'a University, Sana'a, Yemen (hadeelshayef@gmail.com).
24. Ashjan Nasser Abdurabu Bamarhool, MBBS, Ibn Sina National College, Jeddah, Saudi Arabia (toasl222@gmail.com).
25. Manal Jamal Qasim Al-Dhaheri, Faculty of Medicine, Sana'a University, Sana'a, Yemen (manaldhaheri97@gmail.com).
26. Suhaib Hassan Mohammed Yousif, MBBS, Almughtaribeen University, Khartoum, Sudan (sohaibalrome12@gmail.com).
27. Waheeb Ali Al-Garadi, Faculty of Medicine, Thamar University, Thamar City, Yemen (Waheeb99944@gmail.com).
28. Raghad Sameer Qazzaz, MD, Faculty of Medicine, Mutah University, Karak, Jordan (raghadsameer76@gmail.com).
29. Nora Ra'ed Abdullah Atiah, Sana'a University, Faculty of Medicine and Health Sciences, Sana'a, Yemen (atiahnora11@gmail.com).
30. Dua Hassan, Faculty of Medicine, Sana'a University, Sana'a, Yemen (doctorduaa331@gmail.com).
31. Ali Haj Yehia, Faculty of Medicine, Damascus University, Damascus, Syria (alihajyehia45@gmail.com).
32. Tahany A. Qashwa, Faculty of Medicine, Sana'a University, Sana'a, Yemen (tahanyahmed2080@gmail.com).
33. Imen Achouri, Department of Physical Education and Sports Science, Higher Institute of Sport and Physical Education of Sfax, University of Sfax, Tunisia (imenachouri2021@gmail.com).
34. Aseela Reyadh Abdu Mokbel Alhammadi, Faculty of Medicin, Sana'a University, Sana'a, Yemen (aseelaalhammadi78@gmail.com).
35. Lana Sheet, Faculty of Medicine, Aleppo University, Aleppo, Syria (Lanasheitt3@gmail.com).
36. Wehba Hraiz, Faculty of Medicine, Damascus University, Damascus, Syria (hraizwehba@gmail.com).
37. Mohammed Ahmed Salah Mohammed Ahmed Elgak, University of Kassala, Faculty of Medicine, Sudan (Mohammedahmed6218@gmail).
38. Sarah Alawi Almaqdi, Faculty of Medicine, Hadramout University, Hadramout, Yemen (sarahalawi505@gmail.com).
39. Hashem Tehsib Ali, Faculty of Medicine, Al-Furat University, Deir ez-Zor, Syria (hashemabuari98@gmail.com).
40. Kareem Fathy Mohamed, Rheumatology Resident, Faculty of Medicine, Al-Azhar University Hospital, New Damietta (Kfathy638@gmail.com).
